# On the Tautomerism of *N*-Substituted Pyrazolones: 1,2-Dihydro-3*H*-pyrazol-3-ones versus 1*H*-Pyrazol-3-ols

**DOI:** 10.3390/molecules23010129

**Published:** 2018-01-09

**Authors:** Eglė Arbačiauskienė, Sonata Krikštolaitytė, Aiva Mitrulevičienė, Aurimas Bieliauskas, Vytas Martynaitis, Matthias Bechmann, Alexander Roller, Algirdas Šačkus, Wolfgang Holzer

**Affiliations:** 1 Department of Organic Chemistry, Kaunas University of Technology, Radvilėnų pl. 19, LT-50254 Kaunas, Lithuania; egle.arbaciauskiene@ktu.lt (E.A.); sonata.krikstolaityte@ktu.lt (S.K.); aiva.mitruleviciene@gmail.com (A.M.); vytas.martynaitis@ktu.lt (V.M.); 2 Institute of Synthetic Chemistry, Kaunas University of Technology, K. Baršausko g. 59, LT-51423 Kaunas, Lithuania; aurimas.bieliauskas@ktu.lt; 3 Institute of Organic Chemistry, Johannes Kepler University Linz, Altenberger Straße 69, A-4040 Linz, Austria; matthias.bechmann@jku.at; 4 X-ray Structure Analysis Centre, Faculty of Chemistry, University of Vienna, Währinger Straße 42, A-1090 Vienna, Austria; alexander.roller@univie.ac.at; 5 Department of Pharmaceutical Chemistry, Faculty of Life Sciences, University of Vienna, Althanstrasse 14, A-1090 Vienna, Austria

**Keywords:** prototropic tautomerism, 1*H*-pyrazol-3-ol, 1,2-dihydro-3*H*-pyrazol-3-ones, NMR (^1^H, ^13^C, ^15^N), solid state NMR, X-ray structure analysis

## Abstract

The tautomerism of 1-phenyl-1,2-dihydro-3*H*-pyrazol-3-one was investigated. An X-ray crystal structure analysis exhibits dimers of 1-phenyl-1*H*-pyrazol-3-ol units. Comparison of NMR (nuclear magnetic resonance) spectra in liquid state (^1^H, ^13^C, ^15^N) with those of “fixed” derivatives, as well as with the corresponding solid state NMR spectra reveal this compound to exist predominantly as 1*H*-pyrazol-3-ol molecule pairs in nonpolar solvents like CDCl_3_ or C_6_D_6_, whereas in DMSO-*d*_6_ the corresponding monomers are at hand. Moreover, the NMR data of different related 1*H*-pyrazol-3-ol derivatives are presented.

## 1. Introduction

Pyrazolones are interesting chemical entities not only due to their importance as building blocks for the synthesis of various bio-active compounds [1,2,3,4,5,6], but also in respect to their capability to prototropic tautomerism and to the—more uncommon—phenomenon of desmotropy [7]. Whereas for 1-substituted 1*H*-pyrazol-5-ols and their tautomers (2-substituted 2,4-dihydro-3*H*-pyrazol-3-ones and 2-substituted 1,2-dihydro-3*H*-pyrazol-3-ones), a considerably large number of experimental and theoretical studies concerning their tautomerism has been published [8,9,10,11,12], there is much less known about their structural isomers with a 1-substituted 1*H*-pyrazol-3-ol motif. The latter compounds are in as much of interest as they can serve as starting materials for further functionalization [13], the construction of anellated systems [14], as well as for the synthesis of biologically active compounds [15,16,17]. Hence, this study is devoted to investigations with 1-substituted 1*H*-pyrazol-3-ols (tautomers to the corresponding 1,2-dihydro-3*H*-pyrazol-3-ones) and some derivatives carrying different substituents at pyrazole C-4.

## 2. Results and Discussion

**Figure 1:**
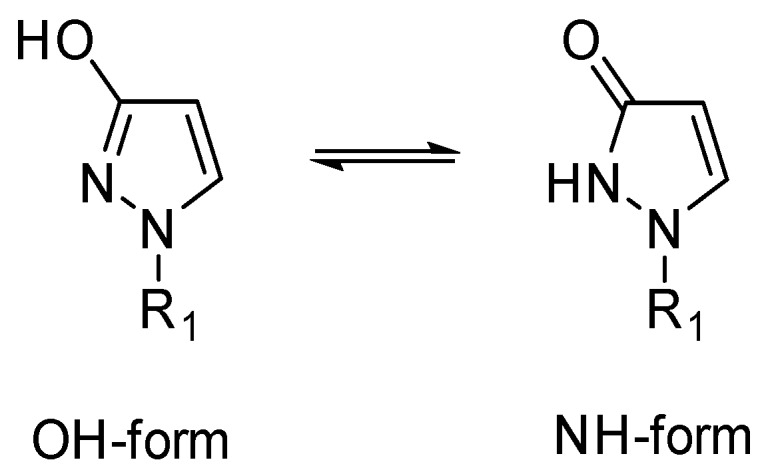
Possible tautomeric forms of 1-substituted 3-hydroxy-1*H*-pyrazoles (1-substituted 1,2-dihydro-3*H*-pyrazol-3-ones).

In principle, for the title compounds, two tautomeric forms are possible, namely the OH-form and the NH-form (Figure 1). In Chemical Abstracts such compounds carrying an alkyl or a (hetero)aryl substituent at the pyrazole nitrogen atom are always listed as 3*H*-pyrazol-3-ones. Hence, as many authors prefer the latter denomination, in the course of this study we investigated which tautomer is really relevant in a solid state and, especially, in solution.

### 2.1. X-ray Analysis of 1-Phenyl-1,2-dihydro-3H-pyrazol-3-one (1-Phenyl-1H-pyrazol-3-ol) *(**1**)*

**Figure 2:**
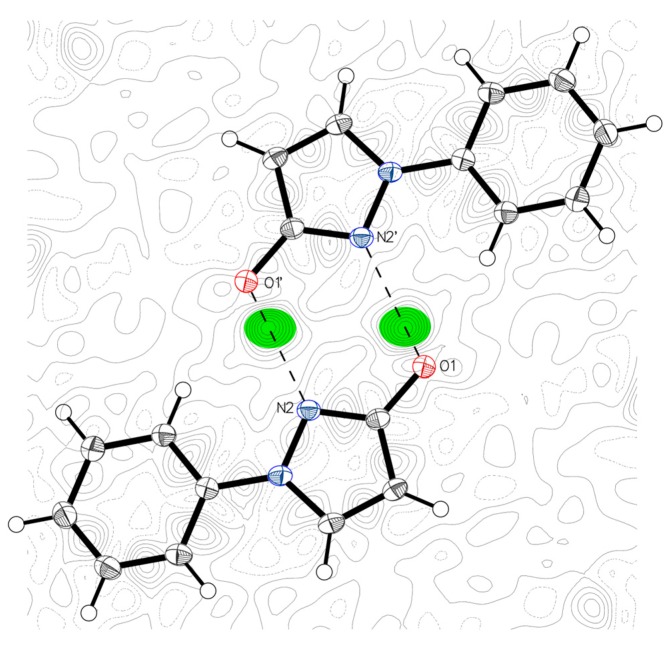
Difference electron density map of **1**. Determination of the H position at O1. The distance of O1 to the electron density position (green shaded) in the direct donor acceptor line is 0.8927 (10) Å. The according distance to N2′ is 1.8339 (10) Å. For further details please follow the CCDC Code.

In the solid state an unambiguous determination of structure and, thus, a safe discrimination between OH and NH-form is possible. In view of this fact crystals of 1-phenyl-1,2-dihydro-3*H*-pyrazol-3-one (1-phenyl-1*H*-pyrazol-3-ol) (**1**)—obtained by crystallization from ethanol/water—were subjected to X-ray structure analysis. It turned out that this compound is present in the 1*H*-pyrazol-3-ol form constituting dimeric units connected by two identical intermolecular hydrogen bonds (Figure 2). In principle, the formation of similar dimeric structures would be also possible by combination of two identical NH-isomers establishing two intermolecular hydrogen bonds between C=O and the NH of the second molecule. However, the electron density map clearly shows the position of the hydrogen atom at the oxygen and thus excludes the latter alternative (Figure 2, further details can be found in the Experimental Section).

### 2.2. Solid State NMR (SSNMR) of ***1***

**Figure 3:**
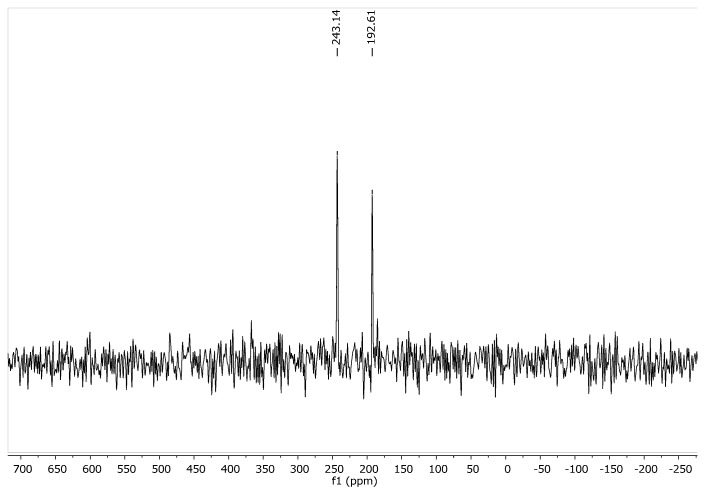
^15^N CP/MAS NMR spectrum of **1**.

The same material as used for the X-ray analysis was subjected to solid state NMR (CP/MAS). As the X-ray analysis revealed the presence of the 1*H*-pyrazol-3-ol form, the solid state NMR spectra also should exclusively origin from this species. Here, due to its simplicity the ^15^N-NMR spectrum is particularly valuable, showing the “pyridine-like” pyrazole N-atom (N-2) at 243.1 ppm, whereas the “pyrrole-like” N-atom (N-1) is resonating at 192.6 ppm referenced against external ^15^NH_4_Cl (39.3 ppm with respect to liquid NH_3_) (Figure 3). The distinct chemical shift difference of N-1 compared to N-2 (Δδ = 50.5 ppm) clearly reflects the fact that the two nitrogen atoms are of different types.

In addition, in ^15^N-NQS (non-quaternary suppression) experiments with different pre-scan dephasing delays (20 µs, 100 µs, 200 µs) the intensities of both ^15^N-NMR resonances remained constant. This behaviour suggests that the two nitrogen atoms are of the same type regarding their protonation status what is only the case for the OH isomer.

### 2.3. NMR Spectra in Solution

Whereas in the solid state, an unambiguous determination of individual tautomeric forms is smoothly possible by X-ray structure analysis, the situation in solution is much more complex. Here, depending on a plethora of different influencing variables, tautomeric equilibria with the simultaneous presence of several tautomeric forms are possible, whereas time-averaged signals are obtained in case of fast exchange. Amongst the appropriate methods for investigating such tautomeric equilibria in solution NMR spectroscopic methods play a prominent role [8,9,18,19,20]. A frequently used concept is the comparison of the data obtained in solution with those of “fixed” derivatives (representing the individual “frozen” tautomeric forms) or with the data of the individual tautomeric forms obtained from solid state NMR experiments. Although this approach comes along with some difficulties (i.e. estimating the difference between the tautomer and the model compound) in many cases fairly good results can be obtained, particularly when one tautomeric form is strongly dominating. However, in cases when several forms are present to a significant extent the precise determination of the percentage composition by interpolation is difficult.

**Figure 4:**
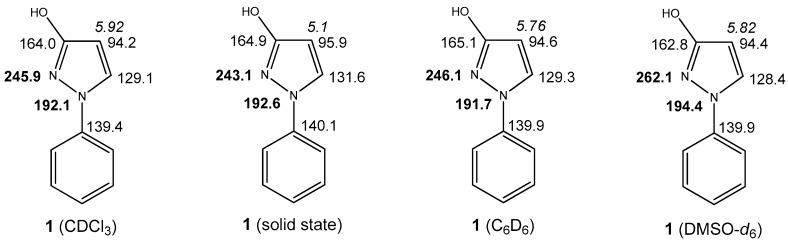
Crucial chemical shifts of **1** in solid state and in different solvents. ^1^H-NMR chemical shifts are represented in italics, ^13^C-NMR chemical shifts in plain text, ^15^N-NMR chemical shifts in bold.

As compound **1** is present as 1*H*-pyrazol-3-ol in the solid state, a comparison of the crucial SSNMR chemical shifts with those in solution should provide valuable information. As outlined in Figure 4, the ^13^C and the ^15^N chemical shifts at the pyrazole nucleus show a high degree of accordance between the solid state and those in CDCl_3_ or in C_6_D_6_ solution which leads to the conclusion that the 3-hydroxy form is far dominating in these solvents.

**Figure 5:**
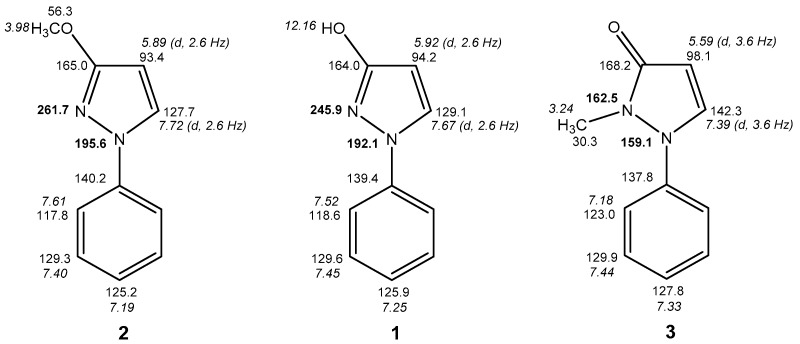
^1^H-NMR (in italics), ^13^C-NMR, and ^15^N-NMR (in bold) chemical shifts of **1** and its “fixed” derivatives **2** and **3** (in CDCl_3_).

In DMSO-*d*_6_ solution it is noticeable that the signal of pyrazole N-2 is clearly shifted downfield compared to the recordings in CDCl_3_ or C_6_D_6_. A possible explanation for this phenomenon is the fact that in the latter nonpolar solvents **1** is obviously present as a dimer of 1*H*-pyrazol-3-ols (like in the solid state) and, thus, the pyrazole N-2 atom is involved in an intramolecular hydrogen bond, whereas—in contrast—in the strong acceptor solvent DMSO-*d*_6_ these intermolecular hydrogen bonds are broken and now monomers are dominating. It is well-known that involvement of a nitrogen’s lone-pair in hydrogen bonds (or—to a larger extent—oxidation, alkylation, or complexation) leads to a marked upfield shift of the corresponding ^15^N resonance [21,22,23]. In 3-methoxy-1-phenyl-1*H*-pyrazole (**2**) (Figure 5) the above mentioned dimerization and, thus, participation of pyrazole N-2 into intermolecular hydrogen bonding is not possible, what is reflected by a larger chemical shift of the latter. Hence, δ(N-2) (261.7 ppm) is now comparable to the value in DMSO-*d*_6_ (262.1 ppm), whereas δ(N-1) in compound **2** (195.6 ppm) and in **1** in different solvents (191.7–194.5 ppm) is very similar. Hence, it can be concluded that **1** is also present as 1*H*-pyrazol-3-ol in DMSO-*d*_6_ solution, however, not in the dimeric form stabilized by intermolecular hydrogen bonds.

In addition, employing the concept of the fixed derivatives we compared the ^1^H-, ^13^C- and ^15^N-NMR chemical shifts, as well as characteristic spin coupling constants of **1** with its *O*-methyl (**2**) and *N*-methyl derivative (**3**), the “fixed” OH- and NH-forms, respectively.

From the data given in Figure 5 the above conclusions are confirmed, namely that compound **1**, which in principle is capable of prototropic tautomerism, is predominantly existing as OH-isomer in CDCl_3_ solution. This is supported by the fact that the ^1^H-, ^13^C-, and ^15^N-NMR chemical shifts of **1** resemble closely to those of the fixed *O*-methyl congener **2**. Especially the ^15^N-chemical shifts are valuable indicators, as the *N*-methyl derivative **3** exhibits two sp^3^-type nitrogen atoms with similar ^15^N-NMR chemical shifts, whereas in **1** and **2** the large chemical shift differences between the two nitrogen atoms in the corresponding molecule hint to different types of N-atoms (sp^3^ and sp^2^). For comparison only, with 1-phenylpyrazolidin-3-one, which formally is the dihydro derivative of the NH form of **1**, we found 105.1 ppm for N-1 and 151.8 ppm for N-2 in DMSO-*d*_6_ solution. Moreover, **1** and **2** show equal sizes of the vicinal ^3^*J*(H4,H5) coupling constant at the pyrazole nucleus (2.6 Hz), whereas in **3** this coupling is considerably larger (3.6 Hz) (Figure 5). An additional difference consists in the ^13^C-NMR chemical shift of Ph C-2/6 which is akin in **1** (118.6 ppm) and **2** (117.8 ppm). In contrast, **3** shows a markedly larger chemical shift for these carbon atoms (123.0 ppm) which can be attributed to some distorsion of phenyl and pyrazole ring obviously induced by the sterical hindrance of the N-methyl group [24,25]. Additionally, the large differences in ^13^C-NMR chemical shifts of pyrazole C-5 (**1**: 129.1 ppm, **2**: 127.7 ppm) in comparison to that of **3** (142.3 ppm) provide an extra confirmation.

Moreover, ^1^H-, ^13^C-, and ^15^N-NMR spectra of compound **1** were additionally taken from C_6_D_6_, DMSO-*d*_6_ and CD_3_OD solutions. As all the significant criteria discussed above were almost similar to those in CDCl_3_ solution, it is reasoned that, also in these solvents, the hydroxy form is far more predominant. The regarding data are presented in the Experimental Section.

**Figure 6:**
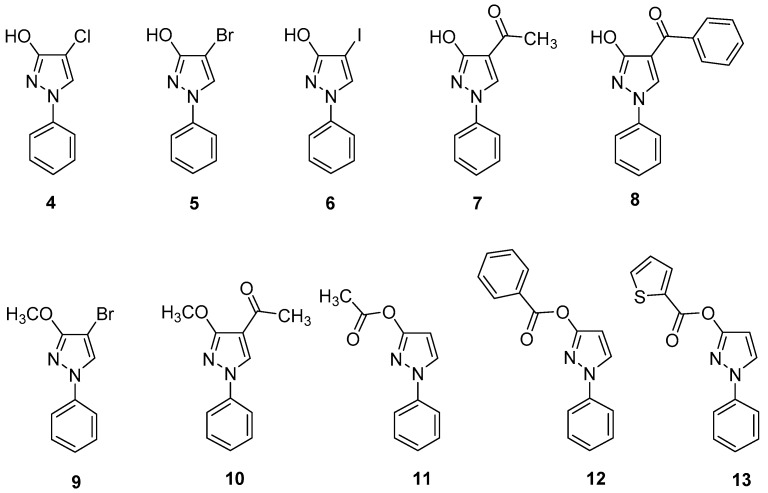
Investigated 4-substituted 1-phenyl-1*H*-pyrazol-3-ols (**4**–**8**) and 3-methoxy congeners (**9**, **10**), as well as 3-*O*-acyl derivatives of **1** (**11**–**13**).

In the following, congeners of **1** carrying a halogen atom or an acyl moiety at pyrazole C-4 were investigated (compounds **4**–**8**). Again, all these species clearly exist as pyrazol-3-ols in CDCl_3_, as well as in DMSO-*d*_6_ solution based on the ^13^C- and ^15^N-NMR chemical shift considerations outlined above. Regarding the 4-bromo derivative **5** a “fixed” 3-methoxy derivative **9** has been described already by us, whose data resemble the “free” 1*H*-pyrazol-3-ol **5** [26]. The same is the case for the pair **7** and **10** (Figure 6). When switching from CDCl_3_ to DMSO-*d*_6_ solution, the ^13^C chemical shift of the carbonyl C-atom in **7** receives an upfield shift of 4.0 ppm (195.7 ppm → 191.7 ppm) which hints to the existence of an intramolecular hydrogen bond—but now—between carbonyl O-atom and OH proton in CDCl_3_ solution, which is broken in the strong acceptor solvent DMSO-*d*_6_ [27]. In principle, also 3-*O*-acyl derivatives of **1** (compounds **11**–**13**) can be regarded as fixed 3-OH derivatives, although the 3-*O*-acyl rest seems to be less comparable to OH than an OCH_3_ group. However, despite larger differences regarding the ^13^C chemical shifts of the pyrazole C-atoms between **1** and **11**–**13** appear, the data of the phenyl ring closely resemble as well as the ^3^*J*(H4,H5) coupling constant at the pyrazole nucleus, which for **11**–**13** is the same as in **1** (2.5–2.6 Hz).

**Figure 7:**
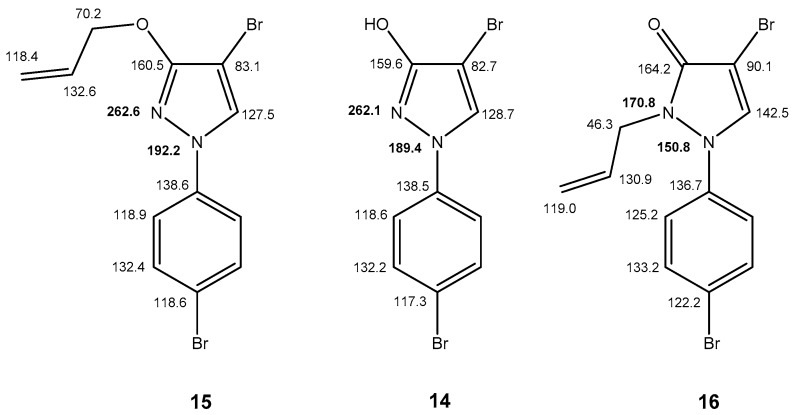
^1^H (in italics), ^13^C and ^15^N (in bold) NMR chemical shifts of **14** and its “fixed” derivatives **15** and **16** (in CDCl_3_).

The triple **14**–**16** provides another example of comparing a free 1*H*-pyrazol-3-ol (**14**) with the corresponding *O*-alkyl (**15**) and *N*-alkyl derivative (**16**), respectively. Again, the selected data depicted in Figure 7 clearly hint that **14** predominantly exists as OH-isomer and not as pyrazol-3-one.

**Figure 8:**
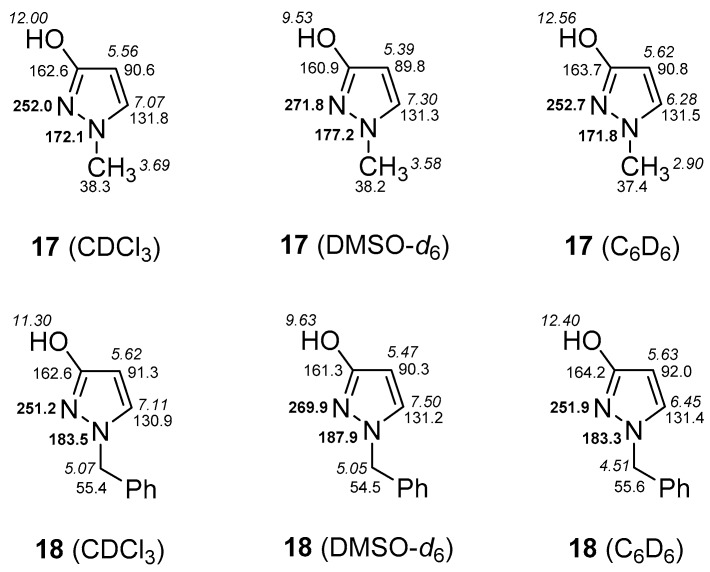
Crucial ^1^H (in italics), ^13^C, and ^15^N (in bold) NMR chemical shifts of 1-methyl-1*H*-pyrazol-3-ol (**17**) and 1-benzyl-1*H*-pyrazol-3-ol (**18**) in CDCl_3_, DMSO-*d*_6_, and C_6_D_6_ solution.

In addition, we investigated 1*H*-pyrazol-3-ols carrying a methyl (**17**) and a benzyl substituent (**18**), respectively, at pyrazole N-1. From the relevant data of these compounds, depicted in Figure 8, the conclusion can be drawn, that also **17** and **18** exist in the 3-hydroxy form in CDCl_3_, DMSO-*d*_6_, and C_6_D_6_ solution. Again, as found with 1-phenyl-1*H*-pyrazol-3-ol **1** and compounds **17**, **18**, the markedly larger chemical shifts of pyrazole N-2 in DMSO-*d*_6_ compared to those in CDCl_3_ or C_6_D_6_ hint to the absence of dimers stabilized by intermolecular hydrogen bonds in this solvent, what is supported by a distinctly smaller ^1^H-NMR chemical shift of the OH-proton in DMSO-*d*_6_ (Figure 8).

## 3. Experimental Section

### 3.1. General Information

Melting points were determined on a Büchi M-565 melting point apparatus (Büchi Labortechnik AG, Flawil, Switzerland) and are uncorrected. IR (infrared) spectra (KBr pellets) were recorded on a Bruker Tensor 27 spectrometer (Bruker Optik GmbH, Ettlingen, Germany) and are reported in wave numbers (cm^−1^). High-resolution ESI-TOF mass spectra were measured on a Bruker maXis spectrometer (Bruker Daltonik GmbH, Bremen, Germany). Elemental analyses were performed at the Microanalytical Laboratory, University of Vienna. ^1^H and ^13^C-NMR spectra were recorded on a Bruker Avance 500 spectrometer (500.13 MHz for ^1^H, 125.77 MHz for ^13^C) (Bruker BioSpin GmbH, Rheinstetten, Germany–valid for all mentioned Bruker NMR spectrometers), a Bruker Avance III 400 spectrometer (400.23 MHz for ^1^H, 100.64 MHz for ^13^C) or a Varian UnityPlus300 spectrometer (299.95 MHz for ^1^H, 75.43 MHz for ^13^C) (Varian, Palo Alto, CA, USA) at 293 K. The centre of the solvent signal was used as an internal standard which was related to TMS with δ 7.26 ppm (^1^H in CDCl_3_), δ 2.49 ppm (^1^H in DMSO-*d*_6_), δ 7.16 ppm (^1^H in C_6_D_6_), δ 3.31 ppm (^1^H in CD_3_OD), δ (δ 77.0 ppm (^13^C in CDCl_3_), δ 39.5 ppm (^13^C in DMSO-*d*_6_), δ 128.06 ppm (^13^C in C_6_D_6_) and δ 49.00 ppm (^13^C in CD_3_OD). The digital resolutions were 0.20 Hz/data point in the ^1^H and 0.33 Hz/data point in the ^13^C-NMR spectra. ^15^N-NMR spectra were obtained on Bruker Avance 500 (50.69 MHz) and Bruker Avance III 400 (40.56 MHz) spectrometers (both equipped with “direct” detection broadband z-gradient observe probes) or on a Bruker Avance III 700 (70.96 MHz) equipped with a 5 mm TCI ^1^H-^13^C/^15^N/D z-gradient cryoprobe, and were measured against external nitromethane (coaxial capillary) and recalculated to liquid ammonia. Solid-state NMR spectra (CP/MAS, MAS: 10 kHz) were recorded on a Bruker Avance III 500 instrument with a broadband MAS-probe for 3.2 mm rotors. CP contact times were 2 ms for (^1^H, ^13^C) and 3 ms for (^1^H, ^15^N). ^1^H RF of 100 kHz was used for spinal64 broadband decoupling. ^1^H, ^13^C-HETCOR spectra were recorded using FSLG homonuclear decoupling during t_1_-evolution and mixing times of 50 µs and 200 µs. ^15^N-NMR spectra were referenced to ^15^NH_4_Cl and recalculated to the liquid ammonia scale (δ ^15^NH_4_Cl 39.3 ppm). ^13^C spectra were referenced to the methylene carbon signals of adamantane and recalculated to the TMS scale (δ ^13^CH_2_ 38.5 ppm). ^1^H chemical shifts were referenced to the NH_3_^+^ resonance in α-Glycine and recalculated to the TMS scale (δ ^15^NH_3_^+^ 8.5 ppm). Product yields were not optimised.

### 3.2. Data of Investigated Compounds 

*1-Phenyl-1H-pyrazol-3-ol* (**1**). Compound **1** was prepared by oxidation of 1-phenylpyrazolidin-3-one with FeCl_3_ according to Reference [28] and recrystallized from EtOH–H_2_O. ^1^H-NMR (CDCl_3_): δ 12.16 (br s, 1H, OH), 7.67 (d, ^3^*J* = 2.6 Hz, 1H, pyrazole H-5), 7.52 (m, 2H, Ph H-2,6), 7.45 (m, 2H, Ph H-3,5), 7.25 (m, 1H, Ph H-4), 5.92 (d, ^3^*J* = 2.6 Hz, 1H, pyrazole H-4). ^13^C-NMR (CDCl_3_): δ 164.0 (^2^*J*(C3,H4) = 2.2 Hz, ^3^*J*(C3,H5) = 10.4 Hz, pyrazole C-3), 139.4 (Ph C-1), 129.6 (Ph C-3,5), 129.1 (^1^*J*(C5,H5) = 187.0 Hz, ^2^*J*(C5,H4) = 8.4 Hz, pyrazole C-5), 125.9 (Ph C-4), 118.6 (Ph C-2,6), 94.2 (^1^*J*(C4,H4) = 180.2 Hz, ^2^*J*(C4,H5) = 7.7 Hz, pyrazole C-4). ^15^N-NMR (CDCl_3_): δ 192.1 (pyrazole N-1), 245.9 (pyrazole N-2). ^1^H-NMR (DMSO-*d*_6_): δ 10.28 (s, 1H, OH), 8.18 (d, ^3^*J* = 2.6 Hz, 1H, pyrazole H-5), 7.67 (m, 2H, Ph H-2,6), 7.40 (m, 2H, Ph H-3,5), 7.15 (m, 1H, Ph H-4), 5.82 (d, ^3^*J* = 2.6 Hz, 1H, pyrazole H-4). ^13^C-NMR (DMSO-*d*_6_): δ 162.8 (^2^*J*(C3,H4) = 2.5 Hz, ^3^*J*(C3,H5) = 10.3 Hz, pyrazole C-3), 139.9 (Ph C-1), 129.4 (Ph C-3,5), 128.4 (^1^*J*(C5,H5) = 188.2 Hz, ^2^*J*(C5,H4) = 8.9 Hz, pyrazole C-5), 124.7 (Ph C-4), 116.8 (Ph C-2,6), 94.4 (^1^*J*(C4,H4) = 178.0 Hz, ^2^*J*(C4,H5) = 8.0 Hz, pyrazole C-4). ^15^N-NMR (DMSO-*d*_6_): δ 194.4 (pyrazole N-1), 262.1 (pyrazole N-2). ^1^H-NMR (C_6_D_6_): δ 12.69 (br s, 1H, OH), 7.33 (m, 2H, Ph H-2,6), 7.06 (m, 2H, Ph H-3,5), 6.97 (d, ^3^*J* = 2.6 Hz, 1H, pyrazole H-5), 6.86 (m, 1H, Ph H-4), 5.76 (d, ^3^*J* = 2.6 Hz, 1H, pyrazole H-4). ^13^C-NMR (C_6_D_6_): δ 165.1 (^2^*J*(C3,H4) = 2.2 Hz, ^3^*J*(C3,H5) = 10.4 Hz, pyrazole C-3), 139.9 (Ph C-1), 129.7 (Ph C-3,5), 129.3 (^1^*J*(C5,H5) = 187.1 Hz, ^2^*J*(C5,H4) = 8.5 Hz, pyrazole C-5), 125.8 (Ph C-4), 118.8 (Ph C-2,6), 94.6 (^1^*J*(C4,H4) = 179.8 Hz, ^2^*J*(C4,H5) = 7.7 Hz, pyrazole C-4). ^15^N-NMR (C_6_D_6_): δ 191.7 (pyrazole N-1), 246.1 (pyrazole N-2). ^1^H-NMR (CD_3_OD): δ 7.89 (^3^*J* = 2.6 Hz, 1H, pyrazole H-5), 7.57 (m, 2H, Ph H-2,6), 7.38 (m, 2H, Ph H-3,5), 7.17 (m, 1H, Ph H-4), 5.82 (d, ^3^*J* = 2.6 Hz, 1H, pyrazole H-4). ^13^C-NMR (CD_3_OD): δ 164.5 (^2^*J*(C3,H4) = 2.6 Hz, ^3^*J*(C3,H5) = 10.2 Hz, pyrazole C-3), 141.3 (Ph C-1), 130.4 (Ph C-3,5), 129.7 (^1^*J*(C5,H5) = 187.6 Hz, ^2^*J*(C5,H4) = 8.6 Hz, pyrazole C-5), 126.3 (Ph C-4), 118.8 (Ph C-2,6), 94.8 (^1^*J*(C4,H4) = 178.6 Hz, ^2^*J*(C4,H5) = 7.9 Hz, pyrazole C-4). ^15^N-NMR (CD_3_OD): δ 193.8 (pyrazole N-1), 256.3 (pyrazole N-2). ^13^C-SSNMR: δ 164.9 (pyrazole C-3), 140.1 (Ph C-1), 132.9, 131.6 (pyrazole C-5), 131.0, 127.1, 118.8, 115.5, 95.9 (pyrazole C-4). ^1^H-SSNMR: δ 11.2 (pyrazole OH), 7.1, 6.9, 6.9, 6.7, 5.8, 5.4 (pyrazole H-5), 5.1 (pyrazole H-4). ^13^C/^1^H-HETCOR-SSNMR: δ 132.9/6.9, 131.6/5.4, 131.0/7.1, 127.1/6.7, 118.8/6.9, 115.5/5.8, 95.9/5.1 (pyrazole C-4). ^15^N-SSNMR: δ 243.1 (pyrazole N-2), 192.6 (pyrazole N-1).

*3-Methoxy-1-phenyl-1H-pyrazole* (**2**) [29]. ^1^H-NMR (CDCl_3_): δ 7.72 (d, ^3^*J* = 2.6 Hz, 1H, pyrazole H-5), 7.61 (m, 2H, Ph H-2,6), 7.40 (m, 2H, Ph H-3,5), 7.19 (m, 1H, Ph H-4), 5.89 (d, ^3^*J* = 2.6 Hz, 1H, pyrazole H-4), 3.98 (s, 3H, OCH_3_). ^13^C-NMR (CDCl_3_): δ 165.0 (^2^*J*(C3,H4) = 2.0 Hz, ^3^*J*(C3,H5) = 10.2 Hz, ^3^*J*(C3,OMe) = 4.0 Hz, pyrazole C-3), 140.2 (Ph C-1), 129.3 (Ph C-3,5), 127.7 (^1^*J*(C5,H5) = 186.2 Hz, ^2^*J*(C5,H4) = 8.3 Hz, pyrazole C-5), 125.2 (Ph C-4), 117.8 (Ph C-2,6), 93.4 (^1^*J*(C4,H4) = 179.2 Hz, ^2^*J*(C4,H5) = 8.1 Hz, pyrazole C-4), 56.3 (^1^*J* = 145.3 Hz, OCH_3_). ^15^N-NMR (CDCl_3_): δ 195.6 (pyrazole N-1), 261.7 (pyrazole N-2).

*2-Methyl-1-phenyl-1,2-dihydro-3H-pyrazol-3-one* (**3**) [30]. ^1^H-NMR (CDCl_3_): δ 7.44 (m, 2H, Ph H-3,5), 7.39 (d, ^3^*J* = 3.6 Hz, 1H, pyrazole H-5), 7.33 (m, 1H, Ph H-4), 7.18 (m, 2H, Ph H-2,6), 5.59 (d, ^3^*J* = 3.6 Hz, 1H, pyrazole H-4), 3.24 (s, 3H, NCH_3_). ^13^C-NMR (CDCl_3_): δ 168.2 (pyrazole C-3), 142.3 (^1^*J*(C5,H5) = 188.5 Hz, ^2^*J*(C5,H4) = 7.7 Hz, pyrazole C-5), 137.8 (Ph C-1), 129.9 (Ph C-3,5), 127.8 (Ph C-4), 123.0 (Ph C-2,6), 98.1 (^1^*J*(C4,H4) = 182.6 Hz, ^2^*J*(C4,H5) = 6.3 Hz, pyrazole C-4), 30.3 (^1^*J* = 140.7 Hz, NCH_3_). ^15^N-NMR (CDCl_3_): δ 159.1 (pyrazole N-1), 162.5 (pyrazole N-2).

*4-Chloro-1-phenyl-1H-pyrazol-3-ol* (**4**) [29]. ^1^H-NMR (CDCl_3_): δ 11.30 (br s, 1H, OH), 7.72 (s, 1H, pyrazole H-5), 7.47 (m, 4H, Ph H-2,3,5,6), 7.30 (m, 1H, Ph H-4). ^13^C NMR (CDCl_3_): δ 159.2 (^3^*J*(C3,H5) = 8.5 Hz, pyrazole C-3), 139.0 (Ph C-1), 129.8 (Ph C-3,5), 126.8 (^1^*J*(C5,H5) = 192.3 Hz, pyrazole C-5), 126.6 (Ph C-4), 118.7 (Ph C-2,6), 98.2 (^2^*J*(C4,H5) = 4.3 Hz, pyrazole C-4). ^15^N-NMR (CDCl_3_): δ 187.6 (pyrazole N-1), 246.5 (pyrazole N-2). ^1^H-NMR (DMSO-*d*_6_): δ 11.02 (s, 1H, OH), 8.52 (s, 1H, pyrazole H-5), 7.66 (m, 2H, Ph H-2,6), 7.43 (m, 2H, Ph H-3,5), 7.20 (m, 1H, Ph H-4). ^13^C-NMR (DMSO-*d*_6_): δ 158.1 (^3^*J*(C3,H5) = 8.5 Hz, pyrazole C-3), 139.3 (Ph C-1), 129.4 (Ph C-3,5), 126.4 (^1^*J*(C5,H5) = 194.4 Hz, pyrazole C-5), 125.2 (Ph C-4), 116.7 (Ph C-2,6), 97.2 (^2^*J*(C4,H5) = 4.6 Hz, pyrazole C-4). ^15^N-NMR (DMSO-*d*_6_): δ 190.0 (pyrazole N-1), 262.0 (pyrazole N-2).

*4-Bromo-1-phenyl-1H-pyrazol-3-ol* (**5**) [29]. ^1^H-NMR (CDCl_3_): δ 11.33 (br s, 1H, OH), 7.73 (s, 1H, pyrazole H-5), 7.48 (m, 4H, Ph H-2,3,5,6), 7.30 (m, 1H, Ph H-4). ^13^C-NMR (CDCl_3_): δ 160.6 (pyrazole C-3, ^3^*J*(C3,H5) = 8.7 Hz), 139.1 (Ph C-1), 129.8 (Ph C-3,5), 129.1 (^1^*J*(C5,H5) = 192.5 Hz, pyrazole C-5), 126.7 (Ph C-4), 118.8 (Ph C-2,6), 82.2 (^2^*J*(C4,H5) = 4.6 Hz, pyrazole C-4). ^15^N-NMR (CDCl_3_): δ 191.7 (pyrazole N-1), 247.9 (pyrazole N-2). ^1^H-NMR (DMSO-*d*_6_): δ 10.99 (s, 1H, OH), 8.51 (s, 1H, pyrazole H-5), 7.67 (m, 2H, Ph H-2,6), 7.43 (m, 2H, Ph H-3,5), 7.21 (m, 1H, Ph H-4). ^13^C-NMR (DMSO-*d*_6_): δ 159.4 (^3^*J*(C3,H5) = 8.9 Hz, pyrazole C-3), 139.3 (Ph C-1), 129.4 (Ph C-3,5), 128.5 (^1^*J*(C5,H5) = 194.7 Hz, pyrazole C-5), 125.3 (Ph C-4), 116.8 (Ph C-2,6), 82.1 (^2^*J*(C4,H5) = 5.2 Hz, pyrazole C-4). ^15^N-NMR (DMSO-*d*_6_): δ 193.7 (pyrazole N-1), 262.5 (pyrazole N-2).

*4-Iodo-1-phenyl-1H-pyrazol-3-ol* (**6**) [29]. ^1^H-NMR (CDCl_3_): δ 11.40 (br s, 1H, OH), 7.72 (s, 1H, pyrazole H-5), 7.48 (m, 4H, Ph H-2,3,5,6), 7.31 (m, 1H, Ph H-4). ^13^C-NMR (CDCl_3_): δ 163.6 (pyrazole C-3, ^3^*J*(C3,H5) = 9.1 Hz), 139.1 (Ph C-1), 133.3 (^1^*J*(C5,H5) = 192.3 Hz, pyrazole C-5), 129.8 (Ph C-3,5), 126.7 (Ph C-4), 118.8 (Ph C-2,6), 46.6 (^2^*J*(C4,H5) = 5.0 Hz, pyrazole C-4). ^15^N-NMR (CDCl_3_): δ 197.2 (pyrazole N-1), 248.1 (pyrazole N-2). ^1^H-NMR (DMSO-*d*_6_): δ 10.89 (s, 1H, OH), 8.40 (s, 1H, pyrazole H-5), 7.67 (m, 2H, Ph H-2,6), 7.41 (m, 2H, Ph H-3,5), 7.18 (m, 1H, Ph H-4). ^13^C-NMR (DMSO-*d*_6_): δ 162.7 (^3^*J*(C3,H5) = 9.4 Hz, pyrazole C-3), 139.3 (Ph C-1), 132.5 (^1^*J*(C5,H5) = 193.8 Hz, pyrazole C-5), 129.4 (Ph C-3,5), 125.2 (Ph C-4), 116.8 (Ph C-2,6), 49.0 (^2^*J*(C4,H5) = 6.3 Hz, pyrazole C-4). ^15^N-NMR (DMSO-*d*_6_): δ 199.3 (pyrazole N-1), 263.0 (pyrazole N-2).

*1-(3-Hydroxy-1-phenyl-1H-pyrazol-4-yl)ethan-1-one* (**7**) [29,31]. ^1^H-NMR (CDCl_3_): δ 9.40 (s, 1H, OH), 8.13 (s, 1H, pyrazole H-5), 7.67 (m, 2H, Ph H-2,6), 7.46 (m, 2H, Ph H-3,5), 7.33 (m, 1H, Ph H-4), 2.48 (s, 3H, COCH_3_). ^13^C-NMR (CDCl_3_): δ 195.7 (C=O), 164.0 (^3^*J*(C3,H5) = 8.7 Hz, pyrazole C-3,), 139.0 (Ph C-1), 129.6 (Ph C-3,5), 128.1 (^1^*J*(C5,H5) = 188.0 Hz, pyrazole C-5), 127.4 (Ph C-4), 119.1 (Ph C-2,6), 108.4 (^2^*J*(C4,H5) = 7.8 Hz, ^3^*J*(C4,CH_3_) = 1.6 Hz, pyrazole C-4), 27.0 (^1^*J* = 127.9 Hz, CH_3_). ^15^N-NMR (CDCl_3_): δ 200.4 (pyrazole N-1), 264.2 (pyrazole N-2). ^1^H-NMR (DMSO-*d*_6_): δ 11.10 (s, 1H, OH), 8.84 (s, 1H, pyrazole H-5), 7.80 (m, 2H, Ph H-2,6), 7.48 (m, 2H, Ph H-3,5), 7.29 (m, 1H, Ph H-4), 2.38 (s, 3H, COCH_3_). ^13^C-NMR (DMSO-*d*_6_): δ 191.7 (C=O), 161.4 (^3^*J*(C3,H5) = 9.0 Hz, pyrazole C-3), 138.8 (Ph C-1), 131.2 (^1^*J*(C5,H5) = 191.8 Hz, pyrazole C-5), 129.4 (Ph C-3,5), 126.4 (Ph C-4), 118.0 (Ph C-2,6), 110.8 (^2^*J*(C4,H5) = 6.8 Hz, ^3^*J*(C4,CH_3_) = 1.4 Hz, pyrazole C-4), 28.5 (^1^*J* = 127.4 Hz, CH_3_). ^15^N-NMR (DMSO-*d*_6_): δ 198.5 (pyrazole N-1), 263.1 (pyrazole N-2).

*(3-Hydroxy-1-phenyl-1H-pyrazol-4-yl)(phenyl)methanone* (**8**) [32]. To a stirred suspension of anhydrous aluminum chloride (16.0 g, 0.12 mol) in 20 mL of carbon disulfide, maintained at room temperature with a water bath, a slurry of 1-phenyl-1*H*-pyrazol-3-yl benzoate (**12**) (2.64 g, 10 mmol) in 70 mL of carbon disulfide was added. After the addition was complete, the reaction mixture was refluxed for 8 h. After the solvent was removed under reduced pressure the residual paste was cooled in an ice bath and a solution of 13.3 mL of 6N hydrochloric acid in 33 mL of ice water was added slowly under stirring to decompose the aluminum chloride salts, then the mixture was allowed to stand overnight. The solid was filtered off, washed with water, dried and recrystallized from EtOH to afford 818 mg (31 %) of **8**, m.p. 138–140 °C (EtOH). ^1^H-NMR (CDCl_3_): δ 9.91 (s, 1H, OH), 8.17 (s, 1H, pyrazole H-5), 7.89 (m, 2H, CPh H-2,6), 7.69 (m, 2H, NPh H-2,6), 7.63 (m, 1H, CPh H-4), 7.54 (m, 2H, CPh H-3,5), 7.45 (m, 2H, NPh H-3,5), 7.32 (m, 1H, NPh H-4). ^13^C-NMR (CDCl_3_): δ 191.7 (C=O), 165.6 (^3^*J*(C3,H5) = 8.8 Hz, pyrazole C-3), 139.0 (NPh C-1), 138.0 (CPh C-1), 132.8 (CPh C-4), 129.5 (NPh C-3,5), 129.1 ((^1^*J*(C5,H5) = 189.6 Hz, pyrazole C-5), 128.9 (CPh C-3,5), 128.2 (CPh C-2,6), 127.4 (NPh C-4), 119.2 (NPh C-2,6), 106.9 (^2^*J*(C4,H5) = 8.0 Hz, pyrazole C-4). ^15^N-NMR (CDCl_3_): δ 202.1 (pyrazole N-1), 264.0 (pyrazole N-2). IR (KBr): 1627 (C=O) cm^−1^. MS *m*/*z* (%): 265 ([M + H]^+^, 100). Anal. Calcd. for C_16_H_12_N_2_O_2_: C, 72.72; H, 4.58; N, 10.60. Found: C, 73.01; H, 4.65; N, 10.27.

*4-Bromo-3-methoxy-1-phenyl-1H-pyrazole* (**9**). The synthesis and the ^1^H and ^13^C-NMR spectra of **9** are described in lit. [26]. ^15^N-NMR (CDCl_3_): δ 194.7 (pyrazole N-1), 262.6 (pyrazole N-2).

*1-(3-Methoxy-1-phenyl-1H-pyrazol-4-yl)ethan-1-one* (**10**) [29]. ^1^H-NMR (CDCl_3_): δ 8.25 (s, 1H, pyrazole H-5), 7.63 (m, 2H, Ph H-2,6), 7.44 (m, 2H, Ph H-3,5), 7.28 (m, 1H, Ph H-4), 4.08 (s, 3H, OCH_3_), 2.46 (s, 3H, COCH_3_). ^13^C-NMR (CDCl_3_): δ 192.3 (C=O), 162.6 (^3^*J*(C3,H5) = 8.8 Hz, ^3^*J*(C3,OCH_3_) = 3.8 Hz, pyrazole C-3), 139.1 (Ph C-1), 130.4 (^1^*J*(C5,H5) = 189.4 Hz, pyrazole C-5), 129.5 (Ph C-3,5), 126.8 (Ph C-4), 118.5 (Ph C-2,6), 111.6 (^2^*J*(C4,H5) = 6.6 Hz, ^3^*J*(C4,CH_3_) = 1.4 Hz, pyrazole C-4), 56.6 (^1^*J* = 146.5 Hz, OCH_3_), 29.1 (^1^*J* = 127.8 Hz, CH_3_). ^15^N-NMR (CDCl_3_): δ 200.0 (pyrazole N-1), 262.9 (pyrazole N-2).

*1-Phenyl-1H-pyrazol-3-yl acetate* (**11**) [29]. ^1^H-NMR (CDCl_3_): δ 7.83 (d, ^3^*J* = 2.5 Hz, 1H, pyrazole H-5), 7.62 (m, 2H, Ph H-2,6), 7.42 (m, 2H, Ph H-3,5), 7.26 (m, 1H, Ph H-4), 6.36 (d, ^3^*J* = 2.5 Hz, 1H, pyrazole H-4), 2.32 (s, 3H, COCH_3_). ^13^C-NMR (CDCl_3_): δ 167.9 (^2^*J*(CO;CH_3_) = 7.0 Hz, C=O), 156.4 (^2^*J*(C3,H4) = 1.2 Hz, ^3^*J*(C3,H5) = 10.9 Hz, pyrazole C-3), 139.6 (Ph C-1), 129.4 (Ph C-3,5), 127.7 (^1^*J*(C5,H5) = 188.5 Hz, ^2^*J*(C5,H4) = 8.5 Hz, pyrazole C-5), 126.4 (Ph C-4), 118.6 (Ph C-2,6), 98.8 (^1^*J*(C4,H4) = 189.9 Hz, ^2^*J*(C4,H5) = 8.1 Hz, pyrazole C-4), 20.9 (^1^*J* = 130.3 Hz, CH_3_). ^15^N-NMR (CDCl_3_): δ 202.9 (pyrazole N-1), 263.8 (pyrazole N-2).

*1-Phenyl-1H-pyrazol-3-yl benzoate* (**12**)*.* A solution of **1** (480 mg, 3 mmol), benzoyl chloride (436 mg, 3.1 mmol) and pyridine (0.1 mL) in toluene (4 mL) was heated at 100 °C for 30 minutes. Then the reaction mixture was poured into water (10 mL), the precipitate was filtered off, washed with water and recrystallized from 50% aqueous ethanol to afford 490 mg (62%) of **11** as colourless crystals, m.p. 59–60 °C. IR (KBr): 1745 (C=O) cm^−1^. ^1^H-NMR (CDCl_3_): δ 8.26 (m, 2H, CPh H-2,6), 7.90 (d, ^3^*J* = 2.6 Hz, 1H, pyrazole H-5), 7.67 (m, 2H, NPh H-2,6), 7.64 (m, 1H, CPh H-4), 7.51 (m, 2H, CPh H-3,5), 7.45 (m, 2H, NPh H-3,5), 7.28 (m, 1H, NPh H-4), 6.52 (d, ^3^*J* = 2.6 Hz, 1H, pyrazole H-4). ^13^C-NMR (CDCl_3_): δ 163.8 (C=O), 156.7 (^2^*J*(C3,H4) = 1.6 Hz, ^3^*J*(C3,H5) = 11.1 Hz, pyrazole C-3), 139.7 (NPh C-1), 133.8 (CPh C-4), 130.4 (CPh C-2,6), 129.4 (NPh C-3,5), 128.8 (CPh C-1), 128.6 (CPh C-3,5), 127.8 (^1^*J*(C5,H5) = 188.5 Hz, ^2^*J*(C5,H4) = 8.6 Hz, pyrazole C-5), 126.5 (NPh C-4), 118.7 (NPh C-2,6), 99.1 (^1^*J*(C4,H4) = 184.1 Hz, ^2^*J*(C4,H5) = 8.0 Hz, pyrazole C-4). ^15^N-NMR (CDCl_3_): δ 203.2 (pyrazole N-1), 277.7 (pyrazole N-2). MS *m*/*z* (%): 265 ([M + H]^+^, 100). Anal. Calcd. for C_16_H_12_N_2_O_2_: C, 72.72; H, 4.58; N, 10.60. Found: C, 72.93; H, 4.45; N, 10.34.

*1-Phenyl-1H-pyrazol-3-yl thiophene-2-carboxylate* (**13**)*.* A solution of **1** (3.20 g, 20 mmol) and 2-thiophenecarbonyl chloride (2.93 g, 20 mmol) in dry toluene (25 mL) was refluxed for 3.5 h. The reaction mixture was poured into water (40 mL), the phases were separated and the aqueous phase was extracted with toluene (2 × 15 mL). The combined organic phases were dried (Na_2_SO_4_) and, after filtration, evaporated under reduced pressure. The residue was recrystallized from EtOH-H_2_O to afford 3.54 g (65%) of **13** as almost colorless crystals, m.p. 62–63 °C. IR (KBr): 1736 (C=O) cm^−1^. ^1^H-NMR (CDCl_3_): δ 8.02 (dd, ^3^*J*(H3,H4) = 3.8 Hz, ^4^*J*(H3,H5) = 1.1 Hz, 1H, Th H-3), 7.88 (d, ^3^*J* = 2.6 Hz, 1H, pyrazole H-5), 7.67 (dd, ^3^*J*(H5,H4) = 4.9 Hz, ^4^*J*(H5,H3) = 1.3 Hz, 1H, Th H-5), 7.65 (m, 2H, Ph H-2,6), 7.43 (m, 2H, Ph H-3,5), 7.27 (m, 1H, Ph H-4), 7.16 (dd, ^3^*J*(H4,H3) = 3.8 Hz, ^3^*J*(H4,H5) = 4.9 Hz, 1H, Th H-4), 6.49 (d, ^3^*J* = 2.6 Hz, pyrazole H-4). ^13^C-NMR (CDCl_3_): δ 159.1 (C=O), 156.2 (^2^*J*(C3,H4) = 1.5 Hz, ^3^*J*(C3,H5) = 11.1 Hz, pyrazole C-3), 139.6 (NPh C-1), 135.1 (^1^*J* = 171.3 Hz, ^2^*J* = 5.6 Hz, ^3^*J* = 9.2 Hz, Th C-3), 134.1 (^1^*J* = 185.7 Hz, ^2^*J* = 7.3 Hz, ^3^*J* = 11.2 Hz, Th C-5), 131.9 (^2^*J* = 5.8 Hz, ^3^*J*(C2,H4) = 9.7 Hz, ^3^*J*(C3,H5) = 5.8 Hz, Th C-2), 129.3 (Ph C-3,5), 128.0 (^1^*J* = 170.6 Hz, ^2^*J*(C4,H3) = 5.0 Hz, ^2^*J*(C4,H5) = 4.0 Hz, Th C-4), 127.7 (^1^*J*(C5,H5) = 188.6 Hz, ^2^*J*(C5,H4) = 8.5 Hz, pyrazole C-5), 126.4 (Ph C-4), 118.6 (Ph C-2,6), 99.0 (^1^*J*(C4,H4) = 184.4 Hz, ^2^*J*(C4,H5) = 8.1 Hz, pyrazole C-4). ^15^N-NMR (CDCl_3_): δ 203.3 (pyrazole N-1), 277.8 (pyrazole N-2). MS *m*/*z* (%): 271 ([M + H]^+^, 100). Anal. Calcd. for C_14_H_10_N_2_O_2_S: C, 62.21; H, 3.73; N, 10.36. Found: C, 62.51; H, 4.01; N, 9.97.

*4-Bromo-1-(4-bromophenyl)-1H-pyrazol-3-ol* (**14**). The synthesis and spectral data of **14** are given in [33].

*Preparation of compounds* **15** *and* **16***.* 4-Bromo-1-(4-bromophenyl)-1*H*-pyrazol-3-ol **14** (1.0 g, 3.15 mmol) was dissolved in DMF (20 mL) and potassium hydroxide (204 mg, 3.65 mmol) was added to the solution. The mixture was stirred for 15 min, then allyl bromide (442 mg, 3.65 mmol) was added, and stirring was continued for 30 min. The reaction mixture was poured into water (40 mL) and extracted with ether (3 × 30 mL). The combined organic extracts were dried over anhydrous sodium sulphate, the solvent was then evaporated under reduced pressure, and the residue subjected to column chromatography (silica gel, eluent: hexane–ethyl acetate 3:1) to yield 824 mg (73%) of compound **15** (R*_f_* 0.68) and 58 mg (5%) of compound **16** (R*_f_* 0.37) as oily substances.

*4-Bromo-1-(4-bromophenyl)-3-(prop-2-en-1-yloxy)-1H-pyrazole* (**15**)*.* ^1^H-NMR (CDCl_3_): δ 7.74 (s, 1H, pyrazole H-5), 7.51 (m, 2H, Ph H-2,6), 7.42 (m, 2H, Ph H-3,5), 6.12 (m, 1H, CH=CH_2_), 5.46 (dd, ^2^*J* = 1.4 Hz, ^3^*J* = 17.2 Hz, 1H, CH=CH_2_(trans)), 5.31 (^2^*J* = 1.4 Hz, ^3^*J* = 10.5 Hz, 1H, CH=CH_2_(cis)), 4.83 (m, OCH_2_). ^13^C-NMR (CDCl_3_): δ 160.5 (^3^*J*(C3,H5) = 5.1 Hz, ^3^*J*(C3,OCH_2_) = 2.8 Hz, pyrazole C-3), 138.6 (Ph C-1), 132.6 (CH=CH_2_), 132.4 (Ph C-3,5), 127.5 (^1^*J* = 191.9 Hz, pyrazole C-5), 118.9 (Ph C-2,6), 118.6 (Ph C-4), 118.4 (CH=CH_2_), 83.1 (^2^*J*(C4,H5) = 5.1 Hz, pyrazole C-4), 70.2 (OCH_2_). ^15^N-NMR (CDCl_3_): δ 192.2 (pyrazole N-1), 262.6 (pyrazole N-2). MS *m*/*z* (%): 361/359/357 ([M + H]^+^, 49/100/51). Anal. Calcd. for C_12_H_10_Br_2_N_2_O: C, 40.26; H, 2.82; N, 7.82. Found: C, 40.40; H, 2.90; N 7.69.

*4-Bromo-1-(4-bromophenyl)-2-(prop-2-en-1-yl)-1,2-dihydro-3H-pyrazol-3-one* (**16**). ^1^H-NMR (CDCl_3_): δ 7.58 (m, 2H, Ph H-3,5), 7.52 (s, 1H, pyrazole H-5), 7.08 (m, 2H, Ph H-2,6), 5.63 (m, 1H, CH=CH_2_), 5.09 (m, 1H, CH=CH_2_(cis)), 4.91 (m, 1H, CH=CH_2_(trans)), 4.34 (m, NCH_2_). ^13^C-NMR (CDCl_3_): δ 164.2 (pyrazole C-3), 142.5 (^1^*J* = 193.9 Hz, pyrazole C-5), 136.7 (Ph C-1), 133.2 (Ph C-3,5), 130.9 (CH=CH_2_), 125.2 (Ph C-2,6), 122.2 (Ph C-4), 119.0 (CH=CH_2_), 90.1 (^2^*J*(C4,H5) = 3.0 Hz, pyrazole C-4), 46.3 (NCH_2_). ^15^N-NMR (CDCl_3_): δ 150.8 (pyrazole N-1), 170.8 (pyrazole N-2). IR (KBr): 1659 (C=O) cm^−1^. MS *m*/*z* (%): 361/359/357 ([M + H]^+^, 49/100/51). Anal. Calcd. for C_12_H_10_Br_2_N_2_O: C, 40.26; H, 2.82; N, 7.82. Found: C, 40.51; H, 3.15; N, 8.13.

*1-Methyl-1H-pyrazol-3-ol* (**17**) [34]. ^1^H-NMR (CDCl_3_): δ 12.00 (s, 1H, OH), 7.07 (d, ^3^*J* = 2.4 Hz, 1H, pyrazole H-5), 5.56 (d, ^3^*J* = 2.4 Hz, 1H, pyrazole H-4), 3.69 (s, 3H, NCH_3_). ^13^C-NMR (CDCl_3_): δ 162.6 (pyrazole C-3), 131.8 (pyrazole C-5), 90.6 (pyrazole C-4), 38.3 (NCH_3_). ^15^N-NMR (CDCl_3_): δ 172.1 (pyrazole N-1), 252.0 (pyrazole N-2). ^1^H-NMR (DMSO-*d*_6_): δ 9.53 (s, 1H, OH), 7.30 (d, ^3^*J* = 2.2 Hz, 1H, pyrazole H-5), 5.39 (d, ^3^*J* = 2.2 Hz, 1H, pyrazole H-4), 3.58 (s, 3H, NCH_3_). ^13^C-NMR (DMSO-*d*_6_): δ 160.9 (pyrazole C-3), 131.3 (pyrazole C-5), 89.8 (pyrazole C-4), 38.2 (NCH_3_). ^15^N-NMR (DMSO-*d*_6_): δ 177.2 (pyrazole N-1), 271.8 (pyrazole N-2). ^1^H-NMR (C_6_D_6_): δ 12.56 (s, 1H, OH), 6.28 (d, ^3^*J* = 2.4 Hz, 1H, pyrazole H-5), 5.62 (d, ^3^*J* = 2.4 Hz, 1H, pyrazole H-4), 2.90 (s, 3H, NCH_3_). ^13^C-NMR (C_6_D_6_): δ 163.7 (pyrazole C-3), 131.5 (pyrazole C-5), 90.8 (pyrazole C-4), 37.4 (NCH_3_). ^15^N-NMR (C_6_D_6_): δ 171.8 (pyrazole N-1), 252.7 (pyrazole N-2).

*1-Benzyl-1H-pyrazol-3-ol* (**18**) [34]. ^1^H-NMR (CDCl_3_): δ 11.30 (s, 1H, OH), 7.33 (m, 3H, Ph H-3,4,5), 7.25 (m, 2H, Ph H-2,6), 7.11 (d, ^3^*J* = 2.5 Hz, 1H, pyrazole H-5), 5.62 (d, ^3^*J* = 2.5 Hz, 1H, pyrazole H-4), 5.07 (s, 2H, NCH_2_). ^13^C-NMR (CDCl_3_): δ 162.6 (^2^*J*(C3,H4) = 2.2 Hz, ^3^*J*(C3,H5) = 10.1 Hz, pyrazole C-3), 136.0 (Ph C-1), 130.9 (^1^*J*(C5,H5) = 185.8 Hz, ^2^*J*(C5,H4) = 8.2 Hz, ^3^*J*(C5,NCH_2_) = 3.2 Hz, pyrazole C-5), 128.8 (Ph C-3,5), 128.1 (Ph C-4), 127.8 (Ph C-2,6), 91.3 (^1^*J*(C4,H4) = 178.9 Hz, ^2^*J*(C4,H5) = 8.0 Hz, pyrazole C-4), 55.4 (^1^*J* = 139.3 Hz, NCH_2_). ^15^N-NMR (CDCl_3_): δ 183.5 (pyrazole N-1), 251.2 (pyrazole N-2). ^1^H-NMR (DMSO-*d*_6_): δ 9.63 (s, 1H, OH), 7.50 (d, ^3^*J* = 2.3 Hz, 1H, pyrazole H-5), 7.32 (m, 2H, Ph H-3,5), 7.27 (m, 1H, Ph H-4), 7.19 (m, 2H, Ph H-2,6), 5.47 (d, ^3^*J* = 2.3 Hz, 1H, pyrazole H-4), 5.05 (s, 2H, NCH_2_). ^13^C-NMR (DMSO-*d*_6_): δ 161.3 (^2^*J*(C3,H4) = 2.5 Hz, ^3^*J*(C3,H5) = 10.1 Hz, pyrazole C-3), 138.0 (Ph C-1), 131.2 (^1^*J*(C5,H5) = 185.8 Hz, ^2^*J*(C5,H4) = 8.5 Hz, ^3^*J*(C5,NCH_2_) = 3.1 Hz, pyrazole C-5), 128.3 (Ph C-3,5), 127.4 (Ph C-2,6), 127.3 (Ph C-4), 90.3 (^1^*J*(C4,H4) = 176.2 Hz, ^2^*J*(C4,H5) = 8.6 Hz, pyrazole C-4), 54,5 (^1^*J* = 139.2 Hz, NCH_2_). ^15^N-NMR (DMSO-*d*_6_): δ 187.9 (pyrazole N-1), 269.9 (pyrazole N-2). ^1^H-NMR (C_6_D_6_): δ 12.40 (s, 1H, OH), 6.93–7.05 (m, 5H, Ph H), 6.45 (d, ^3^*J* = 2.4 Hz, 1H, pyrazole H-5), 5.63 (d, ^3^*J* = 2.4 Hz, 1H, pyrazole H-4), 4.51 (s, 2H, NCH_2_). ^13^C-NMR (C_6_D_6_): δ 164.2 (^2^*J*(C3,H4) = 2.2 Hz, ^3^*J*(C3,H5) = 10.1 Hz, pyrazole C-3), 137.0 (Ph C-1), 131.4 (^1^*J*(C5,H5) = 185.0 Hz, ^2^*J*(C5,H4) = 8.2 Hz, ^3^*J*(C5,NCH_2_) = 3.1 Hz, pyrazole C-5), 129.2 (Ph C-3,5), 128.4 (Ph C-4), 128.4 (Ph C-2,6), 92.0 (^1^*J*(C4,H4) = 178.2 Hz, ^2^*J*(C4,H5) = 8.1 Hz, pyrazole C-4), 55.6 (^1^*J* = 139.2 Hz, NCH_2_). ^15^N-NMR (C_6_D_6_): δ 183.3 (pyrazole N-1), 251.9 (pyrazole N-2).

### 3.3. X-ray Crystal Structure Analysis

**Figure 9:**
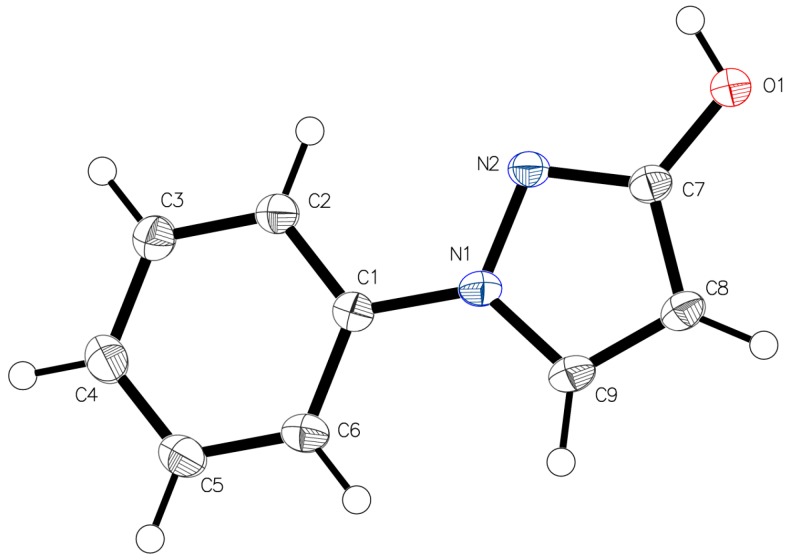
Asymmetric unit of **1**, drawn with 50% displacement ellipsoids.

The X-ray intensity data was measured on a Bruker X8 APEXII equipped with multilayer monochromators, with a Mo K/a INCOATEC micro focus sealed tube, and a Kryoflex II cooling device. The structure was solved by direct methods and refined by full-matrix least-squares techniques. Non-hydrogen atoms were refined with anisotropic displacement parameters. The hydrogen located at O1 was refined without any restraints or constraints. All other hydrogen atoms were inserted at calculated positions and refined with a riding model. The following software was used: Frame integration, Bruker SAINT software package [35] using a narrow-frame algorithm, Absorption correction, SADABS [36], structure solution, SHELXL-2013 [37], refinement, SHELXL-2013 [37], OLEX2 [38], SHELXLE [39], molecular diagrams, OLEX2 [38]*.* Experimental data and CCDC-Code [40] can be found in Table 1. Crystal data, data collection parameters, and structure refinement details are given in Table 2 and Table 3. Molecular structures in “Ortep View” are displayed in Figure 2 and Figure 9. Bond length details are given in Table 4.

The difference electron density map (Figure 2) gives very detailed information about the position of the searched hydrogen atom. The electron density distance of the latter to N2′—as displayed in Figure 2—excludes the possibility of a single bond to the nitrogen for the concerning hydrogen atom. Additionally, its distance to O1 for the electron density proves the position of the hydrogen is located at the oxygen. The free refinement of the hydrogen position without using any restraints or constraints clears all doubts about the non-tautomeric geometry of the molecule in the solid state. Furthermore, at least two very close molecules [41,42] were already measured and interpreted in the same way. In these samples also two identical hydrogen bonds build up molecule pairs because of symmetry reasons.

Pyrazole is well known in crystallography and its different bonds are well characterized by the Handbook of Chemistry and Physics [43]. Table 4 compares the results from compound **1** with corresponding bonds in pyrazole. In detail the double bond N2=C7 in **1** is with 1.329 Å identical to the unweighted mean of the table value for pyrazoles N2=C3 from the Handbook of Chemistry and Physics. The single bond C7-O1 with 1.339 Å is also in strong correlation to expected values like in enols, 1.333 Å, given. Double bond values like in lactams, 1.240 Å, and benzoquinones, 1.222 Å are in contrast to the measured 1.339 Å too small. Finally we proved the position of the searched hydrogen at O1 and we can exclude the NH form in the solid crystalline state.

## Figures and Tables

**Table 1:** Experimental parameter and CCDC-Code.

Sample	Machine	Source	Temp.	Detector Distance	Time/Frame	#Frames	Frame Width	CCDC
	Bruker		[K]	[mm]	[s]		[°]	
**1**	X8	Mo	100	35	25	3739	0.5	1586020

**Table 2:** Sample and crystal data of **1**.

Chemical Formula	C_9_H_8_N_2_O	Crystal System	Orthorhombic	
**Formula weight (g/mol)**	160.17	**Space group**	*Pbca*	
**Temperature (K)**	100	**Z**	8	
**Measurement method**	Φ and ω scans	**Volume (Å^3^)**	1509.4(3)	
**Radiation (Wavelength (Å))**	MoKα (λ = 0.71073)	**Unit cell dimensions (Å) and (°)**	13.6517(13)	90
**Crystal size (mm^3^)**	0.22 × 0.12 × 0.04		6.3663(6)	90
**Crystal habit**	clear colourless plate		17.3673(17)	90
**Density (calculated) (g/cm^3^)**	1.41	**Absorption coefficient/(mm^−1^)**	0.096	
**Abs. correction T_min_**	0.703	**Abs. correction T_max_**	0.746	
**Abs. correction type**	multi-scan	**F(000) (e^−^)**	672	

**Table 3:** Data collection and structure refinement of **1**.

**Index ranges**	−19 ≤ h ≤ 19, −9 ≤ k ≤ 8, −24 ≤ l ≤ 24	**Theta range for data collection (°)**	4.69 to 60.5	
**Reflections number**	62937	**Data/restraints/parameters**	2243/0/113	
**Refinement method**	Least squares	**Final R indices**	all data	R1 = 0.0473, wR2 = 0.1264
**Function minimized**	Σ w(F_o_^2^ − F_c_^2^)^2^		I > 2σ(I)	R1 = 0.0407, wR2 = 0.1202
**Goodness-of-fit on F^2^**	1.085	**Weighting scheme**	w = 1/(σ^2^(F_o_^2^) + (0.0657P)^2^ + 0.6517P)	
**Largest diff. peak and hole (e Å^−3^)**	0.39/−0.21		where P = (F_o_^2^ + 2F_c_^2^)/3	

**Table 4:** Proof of the bond length for the position of H at O1 [43].

Bond Lengths in Crystalline Organic Compounds						Compound 1	
	d	m	σ	q_l_	q_u_		
in pyrazole: (N1–N2)	**1.366**	1.366	0.019	1.350	1.375	N1 N2 single bond	**1.376**
in pyrazole: (N2=C3)	**1.329**	1.331	0.014	1.315	1.339	N1 C7 double bond	**1.329**
in pyrazole: (N1–C5)	**1.357**	1.359	0.012	1.347	1.365	N1 C9 single bond	**1.354**
in enols: C=C–OH	**1.333**	1.331	0.017	1.324	1.342	C7 O1 single bond	**1.339**
in phenols: C_aromatic_-OH	**1.362**	1.364	0.015	1.353	1.373		
in lactams: (C=O)	**1.240**	1.241	0.003	1.237	1.243		
in benzoquinones: (C=O)	**1.222**	1.220	0.013	1.211	1.231		

d is the unweighted mean in Å of all the values for that bond length found in the sample; m is the median in Å of all values; σ is the standard deviation in the sample; q_l_ is the lower quartile for the sample; q_u_ is the upper quartile for the sample.
